# ALKBH5-mediated m6A modification of circCCDC134 facilitates cervical cancer metastasis by enhancing HIF1A transcription

**DOI:** 10.1186/s13046-022-02462-7

**Published:** 2022-08-26

**Authors:** Leilei Liang, Yunshu Zhu, Jian Li, Jia Zeng, Lingying Wu

**Affiliations:** Department of Gynecologic Oncology, National Cancer Center/National Clinical Research Center for Cancer/Cancer Hospital, Chinese Academy of Medical Sciences and Peking Union Medical College, Beijing, 100021 China

**Keywords:** Cervical cancer metastasis, circCCDC134, m6A methylation, p65, miR-503-5p

## Abstract

**Background:**

Metastasis is the main cause of mortality in cervical cancer (CC). Circular RNAs (circRNAs) have been demonstrated to play a crucial role in carcinoma biology. However, the expression and function of circRNAs in cervical cancer metastasis are still unclear.

**Methods:**

In the present study, we identified a circRNA with an N6-methyladenosine (m6A) modification, circCCDC134, whose expression was increased in CC tissues by circRNA-Seq and qPCR. CircCCDC134 upregulation in CC was fine-tuned by ALKBH5-mediated m6A modification, which enhanced its stability in a YTHDF2-dependent manner. The functional experiments illustrated that circCCDC134 enhanced tumour proliferation and metastasis in vitro and in vivo. For the comprehensive identification of RNA-binding proteins, circRNA pull-down and mass spectrometry (ChIRP-MS), chromatin immunoprecipitation-seq (Chip-seq), RNA binding protein immunoprecipitation (RIP) and luciferase reporter assays were used to perform mechanistic investigations.

**Results:**

The results revealed that circCCDC134 recruited p65 in the nucleus and acted as a miR-503-5p sponge to regulate the expression of MYB in the cytoplasm, ultimately stimulating HIF1A transcription and facilitating CC growth and metastasis. Conclusion: These findings indicate that circCCDC134 is an important therapeutic target and provide new regulatory model insights for exploring the carcinogenic mechanism of circCCDC134 in CC.

**Supplementary Information:**

The online version contains supplementary material available at 10.1186/s13046-022-02462-7.

## Background

Cervical cancer (CC) remains the fourth most common gynaecological malignancy worldwide, with approximately 604,000 new cases and 342,000 deaths each year [1]. The clinical outcome of early-stage cervical cancer has improved due to surgery, radiotherapy, and chemotherapy. However, the prognosis of recurrent and metastatic CC is still unsatisfactory, and it is necessary to identify new therapeutic targets to improve the antitumor effect in advanced cervical cancer.

Circular RNAs (circRNAs), as a special type of noncoding RNA, have a closed circular structure without 5′ and 3′ ends and are not degraded by RNA exoenzymes [2]. Based on this circular structure, circRNAs are more conserved and stable. Current research has shown that circRNAs have a high degree of tissue specificity and play an essential role in regulating tumorigenesis and progression [3]. Recent studies have revealed that the known functions of circRNAs include roles as transcriptional regulators, microRNA (miRNA) sponges, RNA-binding protein (RBP) decoys, and translation into proteins [2, 4, 5]. The circ102049-miR-761/miR-192-3p-FRAS1 axis is an antimetastatic target in colorectal cancer [6]; circASAP1 is a key regulator of hepatocellular carcinoma metastasis that acts on miR-326/miR-532-5p-MAPK1/CSF-1 signalling [7]; and circPTK2 inhibits TGF-β-induced EMT and metastasis by controlling TIF1γ in non-small cell lung cancer [8]. However, to date, the expression and regulatory mechanisms of circRNAs in CC metastasis remain far from clear.

Multiple studies have revealed that the N6-methyladenosine (m6A) modification is an important regulator of the production and function of mRNAs and noncoding RNAs [9]. M6A-dependent mRNA modifications modulate RNA stability, splicing, translocation, and nuclear localization, which are vital for cancer development and various biological processes. The process of m6A methylation is reversible and dynamically regulated by methyltransferase complexes (“writers”) and demethylases (“erasers”) and is recognized by m6A binding proteins (“readers”) [10, 11]. Current research shows that there are m6A modifications on circRNAs, and the m6A site is regarded as a trigger initiating circRNA translation [12]. m6A modification of circNSUN2 modulates cytoplasmic export and stabilizes HMGA2 to promote colorectal liver metastasis [13]. circDLC1 is regulated by KIAA1429 (a key component of the m6A methyltransferase complex), and low circDLC1 expression predicts a poor prognosis [14]. However, to the best of our knowledge, the underlying m6A modulator regulatory mechanisms of circRNAs and the functions underlying the mechanisms of circRNAs in CC tumour growth and metastasis are unclear.

In the present study, a circRNA-Seq analysis was performed to identify that hsa_circ_0008806 (a circular RNA derived from CCDC134 termed circCCDC134) is significantly upregulated in CC. Further analyses indicated that m6A modification of circCCDC134 enhanced its stability and facilitated HIF1A transcription, promoting CC tumour growth and metastasis processes by acting as a recruited p65 in the nucleus and as a miR-503-5p sponge to regulate the expression of MYB in the cytoplasm. Thus, this study reveals that circCCDC134 is a novel potential biomarker and therapeutic target in CC.

## Methods

### Tissue samples

Fresh CC tissues were provided by the Cancer Hospital of the Chinese Academy of Medical Sciences. Information regarding the clinical samples is shown in Supplementary Table 1. Experienced pathologists performed the CC diagnosis and disease classification. The study was approved by the Ethics Committee of the Cancer Hospital of the Chinese Academy of Medical Sciences (NCC2021A002).

### Cervical cancer cells and transfection

The human epidermal cell (HUCEC) and cervical cancer cell (ME180, CaSki, SiHA, and HCC94) lines were provided by the Chinese Academy of Sciences Cell Bank (Shanghai, China). All cells were cultured in RPMI-1640 containing 10% foetal bovine serum medium (Invitrogen, Carlsbad, USA). All cells were cultured in a humidified incubator at 37 °C and 5% carbon dioxide.

Small interfering RNAs (siRNAs) targeting the junction sequence of circCCDC134 were designed and synthesized by GenePharma (Shanghai, China). The cells were transfected with these siRNAs with siRNA-mate (GenePharma, Shanghai, China). Forty-eight hours post-infection, the cells were collected and processed for various assays. Lentivirus plasmids of shRNA were constructed by Genechem Company (Shanghai, China) and packaged using pMD2. G and psPAX2 (Addgene, Cambridge, MA) into the cell. For circCCDC134 overexpression (EX-circCCDC134), the sequence was cloned into the pLCDH-ciR vector and packaged using pMD2. G and psPAX2. Information regarding the sequences is shown in Supplementary Table 2.

### Quantitative real-time PCR (qRT–PCR) and immunohistochemistry

The total RNA extraction was performed using RNA-easy Isolation Reagent (No. RC112-01, Vazyme, China). qRT–PCR was performed using a HiScript III 1st Strand cDNA Synthesis Kit (No. R312-01, Vazyme, China) and ChamQ™ Universal SYBR® qPCR Master Mix (No. Q712-02, Vazyme, China) according to the manufacturer’s instructions. For immunohistochemistry, the expression levels of ALKBH5 and HIF1A were measured by immunohistochemical staining with antibodies against ALKBH5 (1:200) (ab195377, Abcam, UK) and HIF1A (1:200) (ab51608, Abcam, UK). The total RNA extraction, qRT–PCR analysis and immunohistochemistry were performed as previously described [15]. Information regarding the sequences is shown in Supplementary Table 2.

### Actinomycin D and RNase R treatment

ME180 cells reached 70% confluence in six-well plates, and the cells were treated with 5 μg/ml actinomycin D and collected at the designated time point. For the RNase R treatment, the total RNA and 2.5 U/μg RNase R (ab286929, Abcam, UK) were incubated at 37 °C for 20 min. After the actinomycin D or RNase R treatment, the RNA expression levels of circCCDC134 and CCDC134 mRNA were analysed by qRT–PCR.

### RNA fluorescence in situ hybridization (FISH) and RNA fractionation assays

RNA-FISH was conducted using a Ribo fluorescence in situ hybridization kit (C10910, RiboBio, China) in accordance with the manufacturer’s directions. circCCDC134 and hsa-miR-503-5p FISH probes were designed and synthesized by RiboBio. In brief, the cells were seeded, fixed with 4% paraformaldehyde and incubated with a hybridized 5 mM probe at 37 °C overnight. All images were visualized and obtained under a confocal microscope (Philips).

In total, 1 × 10^6^ cells were used for the RNA fractionation assays. RNA from the nucleus and cytoplasm was separated by a Cytoplasmic & Nuclear RNA Purification Kit (Norgen Biotek Corp, Canada) following the manufacturer’s instructions.

### RNA pulldown and RNA immunoprecipitation assays

The circRNA pull-down assay was performed with a TRAP (tagged RNA affinity purification) kit (BersinBio, Guangzhou, China) according to the manufacturer’s instructions. In brief, we constructed a plasmid with circCCDC134 and MS2. We also constructed a plasmid with MS2-CP-Flag, which was fused with a mCherry tag (MS2-CP-FlagmCherry). CC cells were transfected with these two plasmids, and circCCDC134 was precipitated by pulldown using anti-Flag antibodies. Lysates derived from cells without the MS2 tagging system were used as controls. The cell lysates were incubated with Protein A/G beads overnight at 4 °C. Then, the RNA and bound proteins were eluted; RNA was analysed by qPCR; and the bound proteins were analysed by label-free mass spectrometry (MS) and Western blot assays.

The RNA immunoprecipitation (RIP) assay was performed with a RIP kit (BersinBio, Guangzhou, China) according to the manufacturer’s instructions. In brief, magnetic beads were incubated with 10 μg of antibodies against ALKBH5 (16837-1-AP, Proteintech, China), YTHDF2 (ab220163, Abcam, Cambridge, USA), p65 (ab16502, Abcam, Cambridge, USA) and normal IgG (Millipore, Massachusetts, USA) overnight at 4 °C. Methylated RNA immunoprecipitation was performed with a MeRIP kit (BersinBio, Guangzhou, China) according to the manufacturer’s instructions, and an anti-N6-methyladenosine (m6A) antibody (ab286164, Abcam, Cambridge, USA) was used for MeRIP. The Western blot and RIP assays were performed as previously described [16].

### Chromatin immunoprecipitation (ChIP) sequencing (ChIP-seq) and CUT&Tag

The ChIP assay was performed with a CUT&Tag Assay Kit (No. TD903-01, Vazyme, China) according to the manufacturer’s instructions. In brief, chromatin solution was added to 10 μg of NF-κB, p65 (8242S, Cell Signaling Technology, China) or Myb antibodies (ab177510, Abcam) and incubated overnight at 4 °C with slow rotation. The ChIP assays were performed as previously described [17].

### Animal studies

The animal experiments were approved by the Animal Center of the Institute of National Cancer Center/Cancer Hospital, CAMS & PUMC and followed the National Institutes of Health Guide for the Care and Use of Laboratory Animals. Four-week-old female nude mice (BALB/c-nu, HFK Bioscience, Beijing, China) were subcutaneously injected with ME180 cells stably expressing shRNA circCCDC134 or an empty vector. The tumour size was measured every 7 days. At the end of the experiment (after 4 weeks), the mice were euthanized by an intraperitoneal injection of 100 mg/kg pentobarbital sodium (Sigma, St. Louis, MO, USA), and the xenograft tumours were resected and weighed. To measure cell metastasis in vivo, a lung metastasis model was used. Briefly, 1 × 10^6^ ME180 cells (shRNA circCCDC134 or empty vector) in 0.1 mL of phosphate-buffered saline were injected into the tail vein of nude mice. After 4 weeks of feeding, the animals were euthanized, and the lung tissues were removed for further processing.

### Statistical analysis

All statistical analyses were performed with GraphPad Prism v. 8.01 (GraphPad Software, La Jolla, CA, USA). Student’s t-test was used to compare values between the test and control groups. *P*-values < 0.05 indicated statistical significance.

### Data availability

The data generated in this study are available upon request from the corresponding author.

## Results

### CircCCDC134 was significantly upregulated in CC tissues

**Fig. 1:**
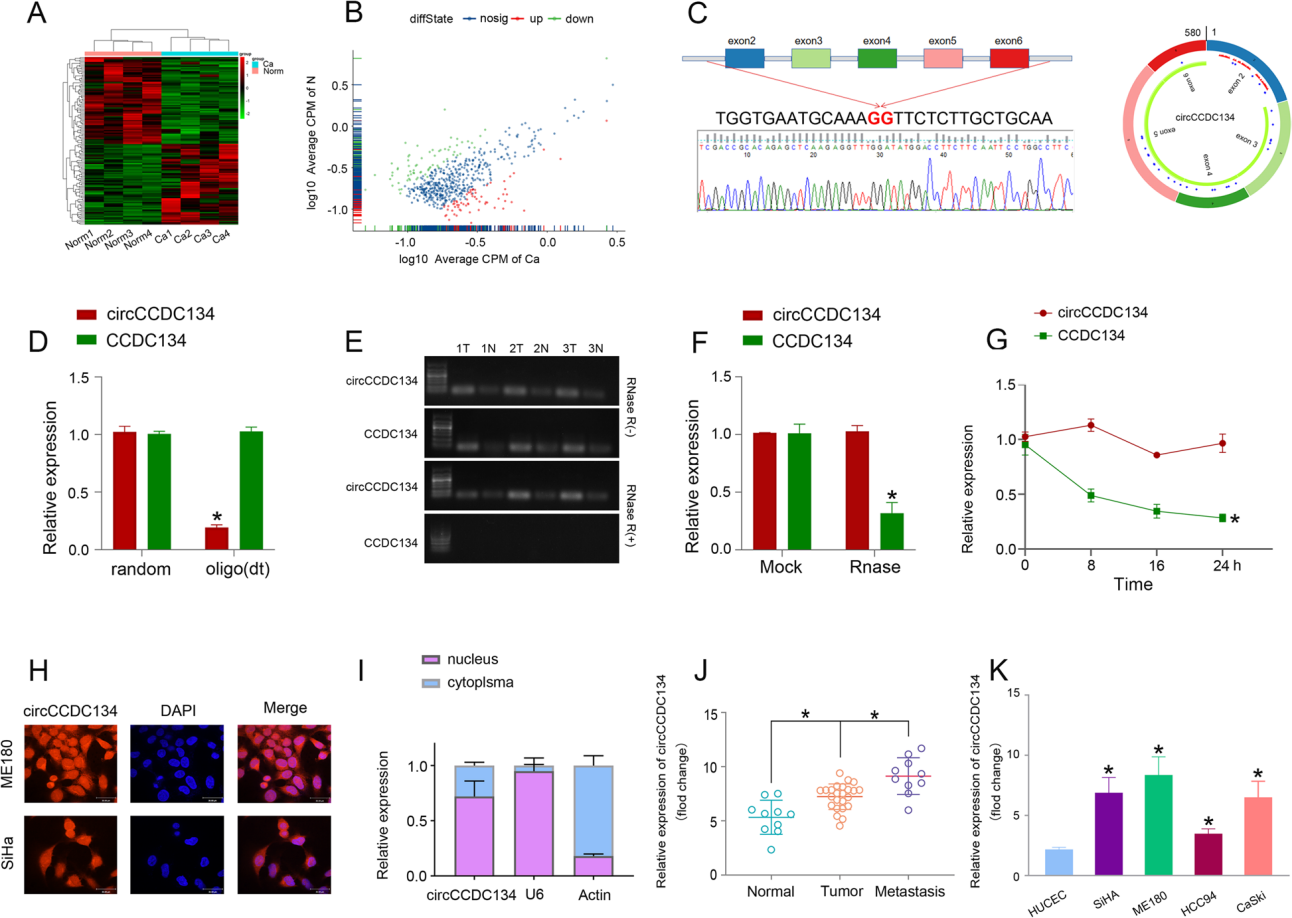
circCCDC134 was significantly upregulated in CC tissues. **A** and **B** Heatmap and scatterplot results showed that there were hundreds of circRNA expression differences in CC tissue. **C** The circular structure of circCCDC134 was confirmed by amplification with divergent primers, followed by Sanger sequencing, which showed that circCCDC134 was generated from exon 2 to exon 6 of the CCDC134 gene through back splicing. **D** We verified the circular feature of circCCDC134 and conducted qRT–PCR using oligo (dT) and random primers, and the results suggested that compared with the random primers, circCCDC134 expression was noticeably reduced, while CCDC134 expression did not change when using the oligo (dT) primers, revealing that circCCDC134 contained no poly-A tail. **E** and **F** RNase R and **G**: actinomycin D suggested that circCCDC134 was more stable than linear CCDC134. **H** and **I** circCCDC134 was found in both the nucleus and cytoplasm via FISH and RNA fractionation assays. **J** and **K** The qRT–PCR results revealed circCCDC134 overexpression in 24 tumour and 10 metastatic tissues and CC cells

circRNA-Seq was used to analyse the dysregulated circRNA expression between 4 pericarcinomatous and 4 CC tissues, which was supported by GENESEED (Guangzhou, China). We found hundreds of circRNAs that varied between the CC tissue and adjacent normal tissue. We detected 21 upregulated circRNAs with a 2-fold change (*p* < 0.05) (Fig. 1A and B), and one of the new molecules that attracted our attention was circCCDC134 (hsa_circ_0008806, 580 bp). Next, the characteristics of circCCDC134 in CC were analysed. Primers for circCCDC134 were designed for the qRT–PCR experiments (Supplementary Fig. 1 A and B). The circular structure of circCCDC134 was confirmed by amplification with divergent primers, followed by Sanger sequencing, which showed that circCCDC134 was generated from exon 2 to exon 6 of the CCDC134 gene through back splicing (Fig. 1C). Oligo (dT) and random primers were employed to identify whether circCCDC134 contains poly-A tails by qRT–PCR. If the expression of the target gene cannot be detected in the reverse transcribed cDNA of Oligo (dT), the RNA has no poly-A tail and is a circular RNA. We verified the circular feature of circCCDC134 and conducted qRT–PCR using oligo (dT) and random primers, and the results suggested that compared with random primers, circCCDC134 expression was noticeably reduced, while CCDC134 expression did not change when using oligo (dT) primers, revealing that circCCDC134 contained no poly-A tail (Fig. 1D). To further detect the features of circCCDC134, both circCCDC134 and the linear mRNA CCDC134 were treated with RNase R and the transcription inhibitor actinomycin D. Ribonuclease R is only able to degrade linear RNA but does not affect circRNA. The results showed that circCCDC134 was more resistant to both RNase R (Fig. 1E and F) and actinomycin D (Fig. 1G), suggesting that circCCDC134 was more stable than linear CCDC134. For the localization of circRNA, FISH and RNA fractionation assays were further performed. The cytoplasmic and nuclear RNA of ME180 and SiHA cell lines were separated and extracted, and qRT–PCR was performed to determine circCCDC134 expression in the cytoplasm and nucleus by RNA fractionation assays. The fluorescence signal (Fig. 1H) and the expression of circCCDC134 (Fig. 1I and Supplementary Fig. 1C) were found in both the nucleus and cytoplasm in the ME180 and SiHA cell lines. qRT–PCR assays were used to explore the expression of circCCDC134 in CC tissues and cells. The qRT–PCR results revealed circCCDC134 overexpression in the tumour and metastatic tissues (Fig. 1J) and CC cells. Among the CC cells, the expression of circCCDC134 was higher in the ME180 and SiHA cells and lower in the HCC94 cells than in the other CC cells (Fig. 1K).

### m6A modification of circCCDC134 enhanced its stability

**Fig. 2:**
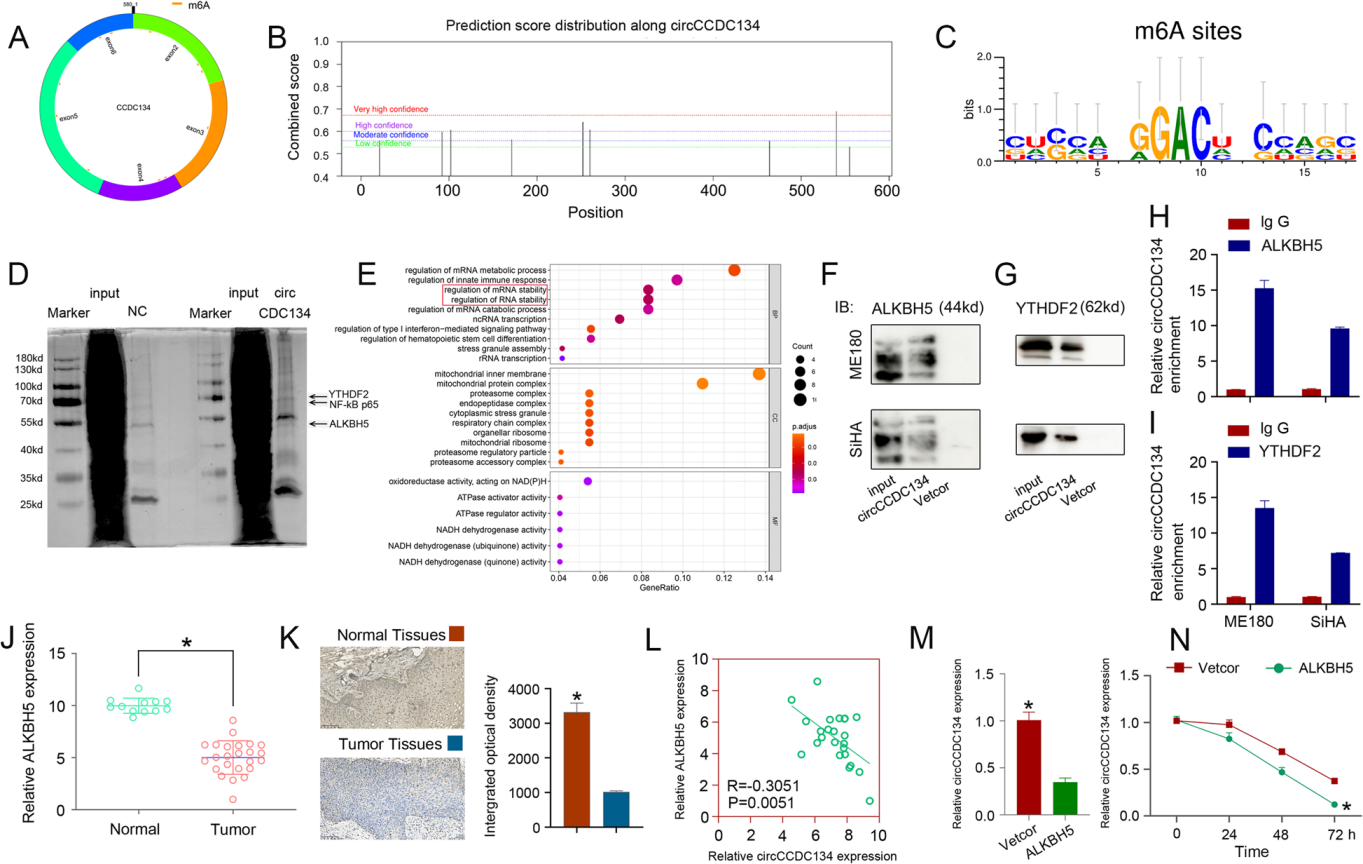
m6A modification of circCCDC134. **A** and **B** m6A modification of circCCDC134 in the circPrimer and SRAMP prediction servers. **C** Motif analysis of the circCCDC134 methylation site. **D** ChIRP-MS showed that circCCDC134 cooperated with ALKBH5 and YTHDF2. **E** The GO results revealed that ALKBH5 and YTHDF2 may affect RNA stability. **F** and **G** ChIRP and **H** and **I**: RIP assays demonstrated that both ALKBH5 and YTHDF2 could interact with circCCDC134. **J** and **K** The qRT–PCR (24 tumour tissues) and IHC (5 pairs of CC tissues) results revealed that the expression of ALKBH5 was low in CC. **L** circCCDC134 expression was significantly negatively correlated with ALKBH5 expression in CC based on qPCR results. **M** and **N** qRT–PCR and actinomycin D assays showed that the expression and stability of circCCDC134 were decreased with ALKBH5 overexpression

First, we explored the potential mechanism of circCCDC134 upregulation in CC. Previous research has shown that m6A modification plays a pivotal role in posttranscriptional regulation and the biogenesis of circRNAs. We analysed the m6A modification of circCCDC134 using circPrimer (Fig. 2A) and the SRAMP prediction server (http://www.cuilab.cn/sramp/) (Fig. 2B), and there were many m6A modification sites of circCCDC134. The motif analysis of the circCCDC134 methylation site based on the very high confidence of SRAMP is shown in Fig. 2C.

To explore this further, we first performed RNA pull-down assays and a mass spectrometry analysis (ChIRP-MS) to screen circCCDC134-interacting proteins and comprehensively identify RNA-binding proteins. The results of ChIRP-MS (Supplementary Fig. 1D and E) and silver staining (Fig. 2D) showed that circCCDC134 cooperated with multiple proteins, including ALKBH5 and YTHDF2. Previous studies demonstrated that a-ketoglutarate-dependent dioxygenase AlkB homologue 5 (ALKBH5) (a demethylase) can remove m6A methylation from its target RNAs and lead to lower levels of m6A [18]; the reader protein YTH N6-methyladenosine RNA binding protein 2 (YTHDF2) can selectively bind m6A-modified RNAs, recruit them to mRNA decay sites and then control target RNA stability [19]. To gain insight into the functions of circCCDC134-binding proteins, we implemented Protein–Protein Interaction Networks (PPI) and Gene Ontology (GO) based on mass spectrometry data. The PPI (Supplementary Fig. 1 F) and GO (Fig. 2E) results revealed that ALKBH5 and YTHDF2 mediated m6A modification, which could affect RNA stability. Therefore, we propose that circCCDC134 stability in CC is tuned by ALKBH5-mediated m6A modification in a YTHDF2-dependent manner, increasing its expression. To further identify the ALKBH5 protein and YTHDF2 protein interacting with circCCDC134, we performed circRNA pull-down and RIP assays. The results of the circCCDC134 pull-down assay showed that the ALKBH5 protein and YTHDF2 protein could bind circCCDC134-MS2 (Fig. 2F and G). We performed RIP assays using anti-ALKBH5 or anti-YTHDF2 antibodies, and the results demonstrated that both ALKBH5 and YTHDF2 could interact with circCCDC134 (Fig. 2H and I). In order to quantify m6A level of circCCDC134, m6A RNA immunoprecipitation (MeRIP) was performed. Gene-specific m6A qPCR to detect the m6A methylation status of circCCDC134 was performed and the results indicated that an reduction of m6A methylation in the ALKBH5 overexpression group of SiHA (Supplementary 1 G) and ME180 (Supplementary 1 H) cell lines. Next, we further confirmed the effect of ALKBH5 expression on circCCDC134 expression. We further analysed the correlation between ALKBH5 and circCCDC134 expression in CC. The qPCR and IHC results showed that the expression of ALKBH5 was low in CC (Fig. 2J and K), and patients with high ALKBH5 expression had much longer overall survival (Supplementary Fig. 1I). Next, circCCDC134 expression was significantly negatively correlated with ALKBH5 expression in CC (Fig. 2L) based on the qPCR results. Moreover, the qRT–PCR and actinomycin D assays showed that the expression (Fig. 2M) and stability (Fig. 2N) of circCCDC134 were decreased with the overexpression of ALKBH5. Taken together, our data suggest that the ALKBH5-mediated m6A methylation of circCCDC134 is responsible for circCCDC134 upregulation by increasing its RNA stability via YTHDF2.

### circCCDC134 facilitates CC cell proliferation and metastasis in vitro and in vivo

**Fig. 3:**
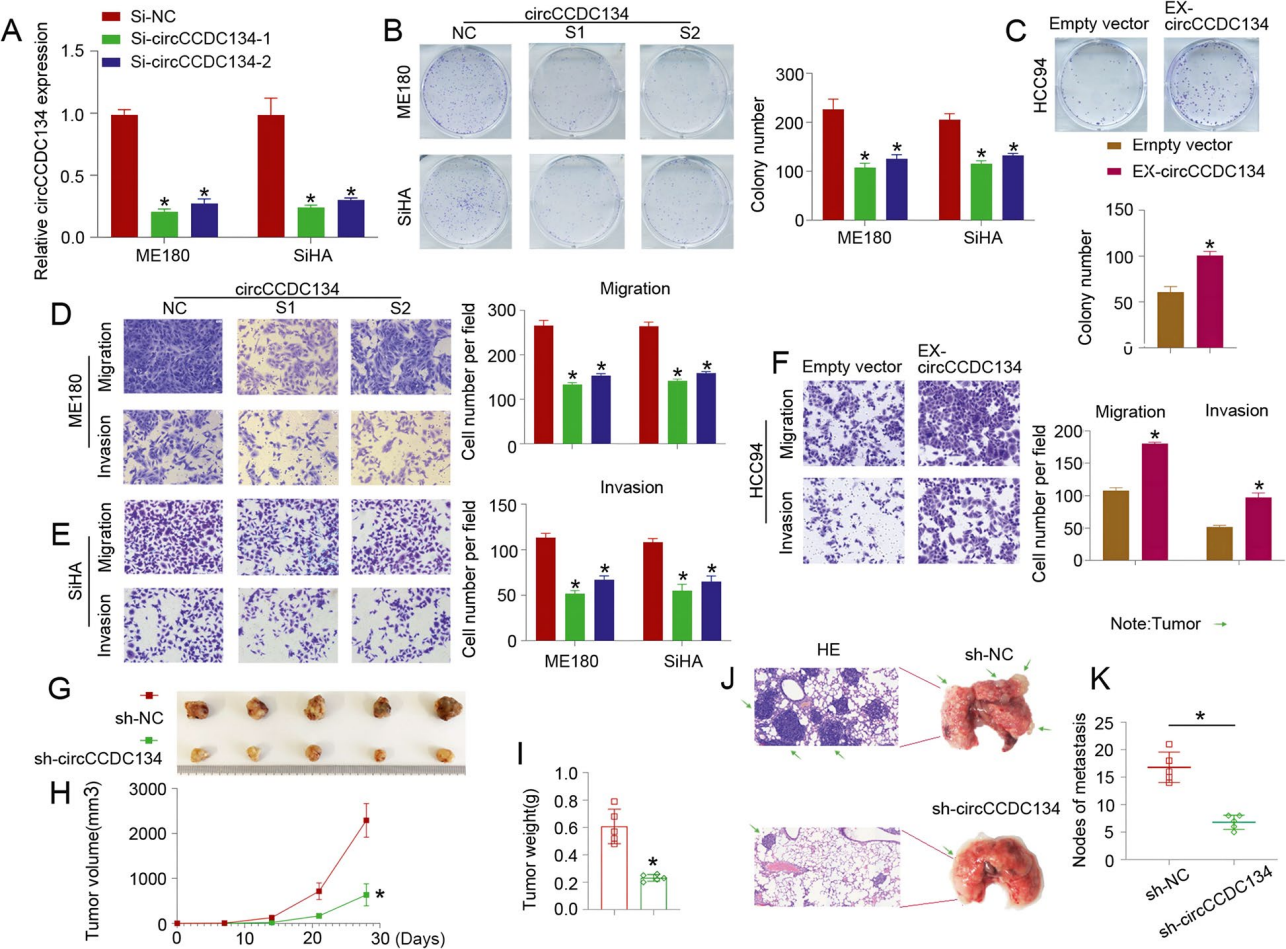
circCCDC134 facilitates CC proliferation and metastasis. **A** The qPCR results showed that Si-circCCDC134-1 and Si-circCCDC134-2 could significantly inhibit circCCDC134 expression in CC cells (ME180 and SiHA cells). **B** and **C** Colony formation and **D**, **E** and **F**: Transwell assays revealed the role of circCCDC134 in promoting the proliferation and metastasis of CC cells. Xenografts formed by ME180 cells bearing sh-circCCDC134 exhibited a significantly slower growth rate (**G** and **H**) and lower tumour weight (**I**) than scrambled controls. In the lung metastasis models (5 nude mice in the sh-NC group and 5 nude mice in the sh-circCCDC134 group), **J** and **H**: the volumes and the number of metastatic nodes were significantly decreased in the sh-circCCDC134 group

To gain insight into the function of circCCDC134, we characterized the oncogenic phenotypes in ME180 and SiHA cells with circCCDC134 silencing (Si-circCCDC134-1 and Si-circCCDC134-2) and HCC94 cells with circCCDC134 overexpression (EX-circCCDC134). The qPCR results showed that Si-circCCDC134-1 and Si-circCCDC134-2 could significantly inhibit circCCDC134 expression in CC cells (ME180 and SiHA cells) (Fig. 3A), and we chose Si-circCCDC134-1 and Si-circCCDC134-2 for the cell function experiments. We investigated the role of circCCDC134 in the proliferation of CC cells by a colony formation assay. To assess the influence of circCCDC134 on cell migration and invasion, Transwell assays were used to detect the cell migration and invasion capacity. The colony formation and Transwell assays revealed that the knockdown of circCCDC134 impaired the proliferation (Fig. 3B), migration (Fig. 3D), and invasion (Fig. 3E) of CC cells. Additionally, the overexpression of circCCDC134 significantly promoted CC cell proliferation (Fig. 3C), migration and invasion (Fig. 3F). To further evaluate the effect of circCCDC134 on CC tumour growth and metastasis in vivo, we established xenograft growth and lung metastasis models. Notably, the xenografts (Fig. 3G) formed by ME180 cells bearing sh-circCCDC134 exhibited significantly slower growth rates (Fig. 3H) and lower tumour weights (Fig. 3I) than the scrambled controls. Furthermore, the volumes of the metastatic nodes in the lung in the circCCDC134 knockdown group were apparently smaller than those in the control group (Fig. 3J), and the number of metastatic nodes was significantly decreased in the circCCDC134 silencing group (Fig. 3K). Taken together, these findings suggest that circCCDC134 acts as an oncogene in CC metastasis by promoting cellular proliferation, migration and invasion.

### circCCDC134 dysregulation contributes to the abnormal expression of genes in CC

**Fig. 4:**
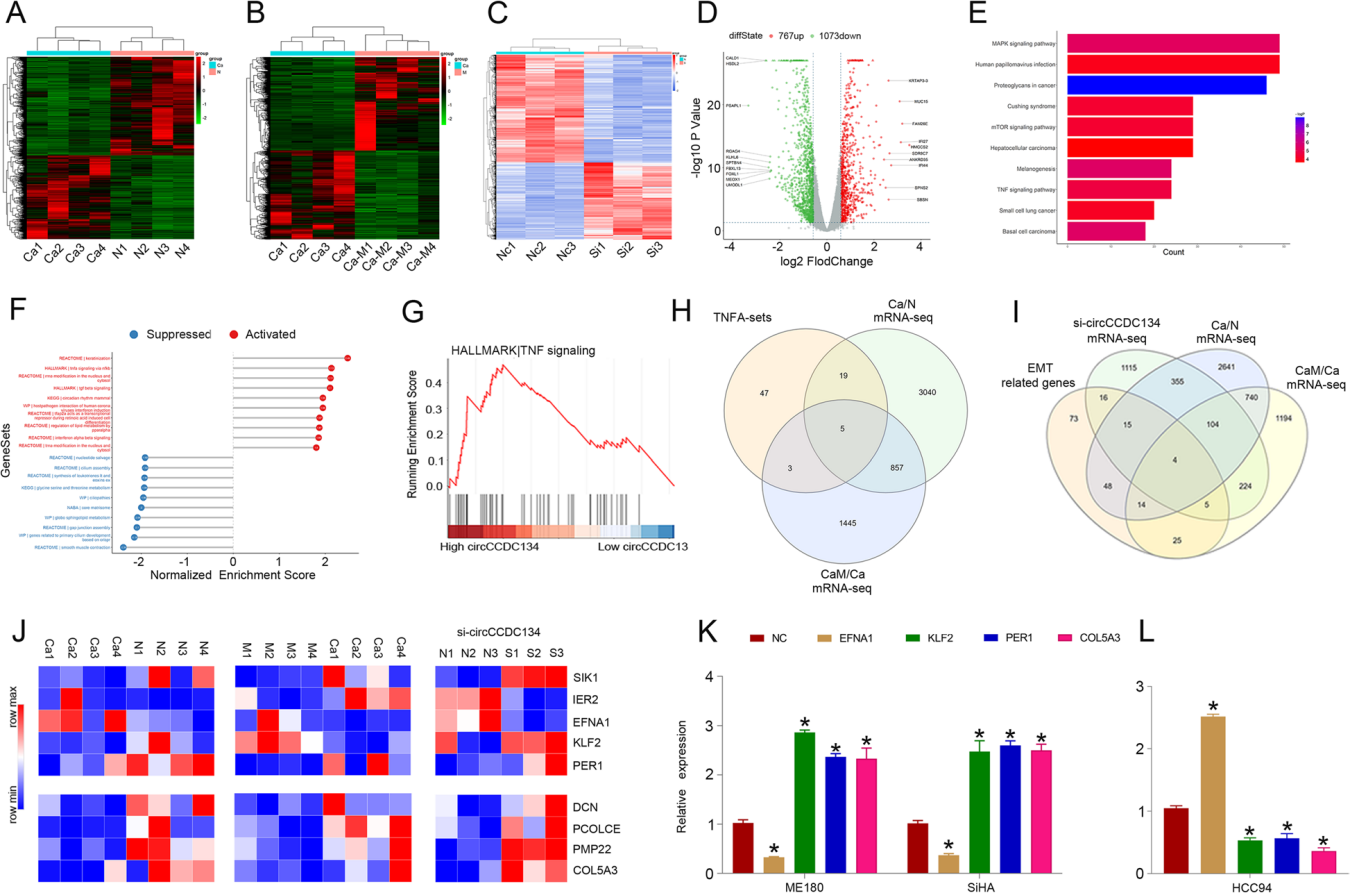
circCCDC134 contributes to abnormal gene expression in CC. **A** and **B** Heatmap of mRNA-seq data of CC and metastatic tissues. **C** and **D** Heatmap and scatterplot results of si-circCCDC134 mRNA-seq data. **E** and **F** KEGG and GSEA based on si-circCCDC134 mRNA-seq data. **G** GSEA of the TNF signalling pathway. **H** and **I** Identification of potential TNF and EMT mRNAs regulated by circCCDC134. **J** Heatmap of TNF and EMT mRNAs regulated by circCCDC134. **K** and **L** The expression of EFNA1 was decreased in ME180 and SiHA cells and increased in HCC94 cells that were induced by circCCDC134. KLF2, PER1 and COL5A3 expression was increased in ME180 and SiHA cells and decreased in HCC94 cells induced by circCCDC134

To clarify the abnormal expression of genes related to CC metastasis and circCCDC134 disorder, mRNA-seq of CC (Fig. 4A), mRNA-seq of metastasis tissues (Fig. 4B) and mRNA-seq of si-circCCDC134/ME180 cells (ME180 cells transfected with si-circCCDC134-1) (Fig. 4C and D) were carried out. Furthermore, we implemented Kyoto Encyclopedia of Genes and Genomes (KEGG) (Fig. 4E) and gene set enrichment analysis (GSEA) (Fig. 4F and G) based on the si-circCCDC134 mRNA-seq data. The results revealed that the upregulated circCCDC134 expression was closely associated with the TNF signalling pathway. It has been acknowledged that the TNF signalling pathway [20] and epithelial-mesenchymal transition (EMT) [21] contribute to the metastatic progression of CC. To identify potential TNF and EMT mRNAs regulated by circCCDC134, we analysed the mRNA-seq data of CC tissues, metastatic tissues and the si-circCCDC134 group. Regarding TNF-related genes, by combining the TNF signalling pathway in the si-circCCDC134, CC and metastasis mRNA-seq data results, 5 genes were found, including SIK1, IER2, EFNA1, KLF2 and PER1 (Fig. 4H and J). Regarding EMT-related genes, 200 EMT-related genes were downloaded from the Molecular Signature database v7.1 (http://www.broad.mit.edu/gsea/msigdb/) [22]. By combining the EMT-related gene data, si-circCCDC134 mRNA-seq data, CC mRNA-seq data and metastasis mRNA-seq data, 4 genes were found, including DCN, PCOLCE, PMP22 and COL5A3 (Fig. 4I and J). Next, we explored the expression and survival analysis of these genes in CC based on TCGA databases, and the analysis revealed that EFNA1, KLF2, PER1 and COL5A3 expression was closely associated with CC overall survival (Supplementary Fig. 2). In addition, the qRT–PCR analysis confirmed that the expression of EFNA1 was decreased in ME180 and SiHA cells and increased in HCC94 cells that were induced by circCCDC134. Regarding KLF2, PER1 and COL5A3, their expression was increased in ME180 and SiHA cells and decreased in HCC94 cells induced by circCCDC134 (Fig. 4K and L). These results demonstrate that circCCDC134 dysregulation contributes to the abnormal expression of TNF-related mRNAs and EMT-related mRNAs.

### circCCDC134 stimulates HIF1A transcription by recruiting p65 and acting as a miR-503-5p sponge

**Fig. 5:**
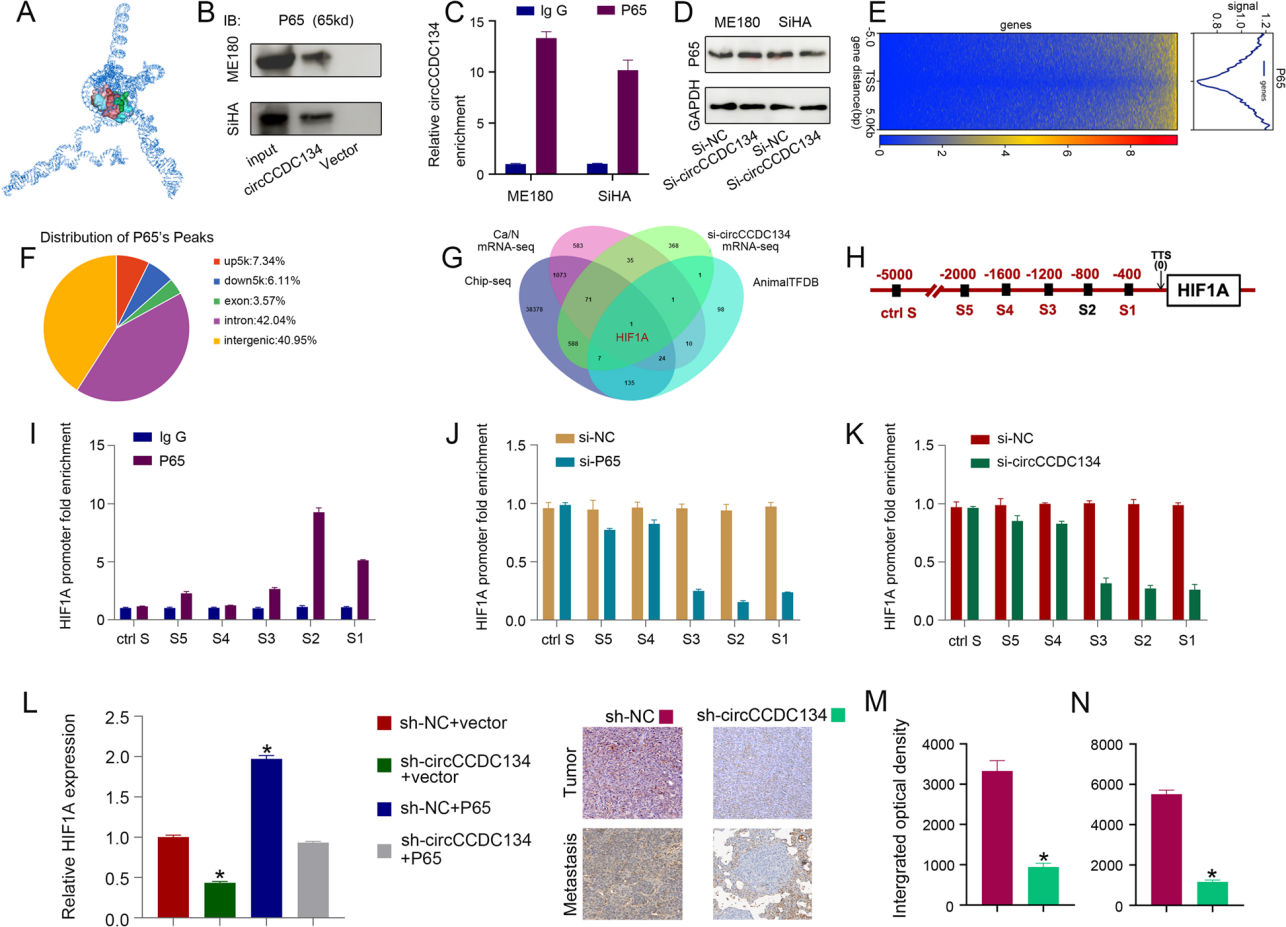
circCCDC134 stimulates HIF1A transcription by recruiting p65. **A** The molecular simulation results supported that circCCDC134 could perfectly dock p65. The results of **B**: circRNA pull-down and **C**: RIP assays demonstrated that p65 could interact with circCCDC134, and **D**: no significant change was found in the p65 protein levels upon circCCDC134 knockdown. **E** Heatmaps and **G**: pie charts of p65 ChIP-seq. HIF1A was found to be the key target gene of p65. **H** and **I** p65 was enriched in the region − 1200 to − 400 bp from the transcription start site of the HIF1A promoter. p65 enrichment of the HIF1A promoter was reduced after interfering with **J**: p65 and **K**: circCCDC134 expression. **L** The expression of HIF1A was positively correlated with the expression of circCCDC134. **M** and **N** Immunohistochemistry showed that HIF1A was positively correlated with the expression of circCCDC134 in the xenograft growth and lung metastasis models

Many studies have demonstrated that circRNAs play regulatory roles by binding proteins and sponging miRNAs. In the above experiments, ChIRP-MS was performed to determine whether circCCDC134 could interact with binding proteins, and the results showed that circCCDC134 cooperated with multiple nuclear proteins. Among the binding proteins, we found that circCCDC134 could interact with p65, which is a very important transcription factor that promotes CC metastasis [23] and interacts with the TNF signalling pathway [24] and EMT [25]. We explored the possibility that circCCDC134 interacts with p65, and the secondary structure of circCCDC134 was assembled in Mfold (version 2.3) following the methods described by Du William W et al. [26]. The molecular simulation results supported that circCCDC134 could perfectly dock with p65 (Fig. 5A). The results of the circRNA pull-down (Fig. 5B) and RIP (Fig. 5C) assays demonstrated that p65 could interact with circCCDC134. The western blot results showed that there was no significant change in the p65 protein levels upon circCCDC134 knockdown, indicating that circCCDC134 does not regulate the expression of the P65 protein (Fig. 5D). Furthermore, ChIP-seq and ChIP–qPCR assays were used to identify target genes regulated by p65. As shown in Fig. 5E and F, p65 binds the upstream 5 k region of 7.34% of genes that contain the gene promoter region based on the ChIP-seq results. Furthermore, AnimalTFDB 3.0, which is a comprehensive resource for the annotation and prediction of animal transcription factors, was used to analyse the key target genes of p65 [27]. By combining AnimalTFDB 3.0 data of p65, si-circCCDC134 mRNA-seq data, CC mRNA-seq data and P65 ChIP-seq data, HIF1A was found (Fig. 5G and H). We tested whether circCCDC134 regulates HIF1A transcription by enhancing p65, and the results were as follows: 1. p65 was enriched in the region − 1200 to − 400 bp from the transcription start site of the HIF1A promoter (Fig. 5I); 2. p65 enrichment at − 1200 to − 400 bp from the transcription start site of the HIF1A promoter was reduced after interference with p65 and circCCDC134 expression (Fig. 5J and K). Moreover, the qPCR results showed that the expression of HIF1A was rescued by transfection with p65 and si-circCCDC134 (Fig. 5L). Immunohistochemistry showed that the expression of HIF1A was positively correlated with the expression of circCCDC134 in the xenograft growth and lung metastasis models (Fig. 5M and N). These results suggest that circCCDC134 interacts with p65 to enhance HIF1A transcription.

**Fig. 6:**
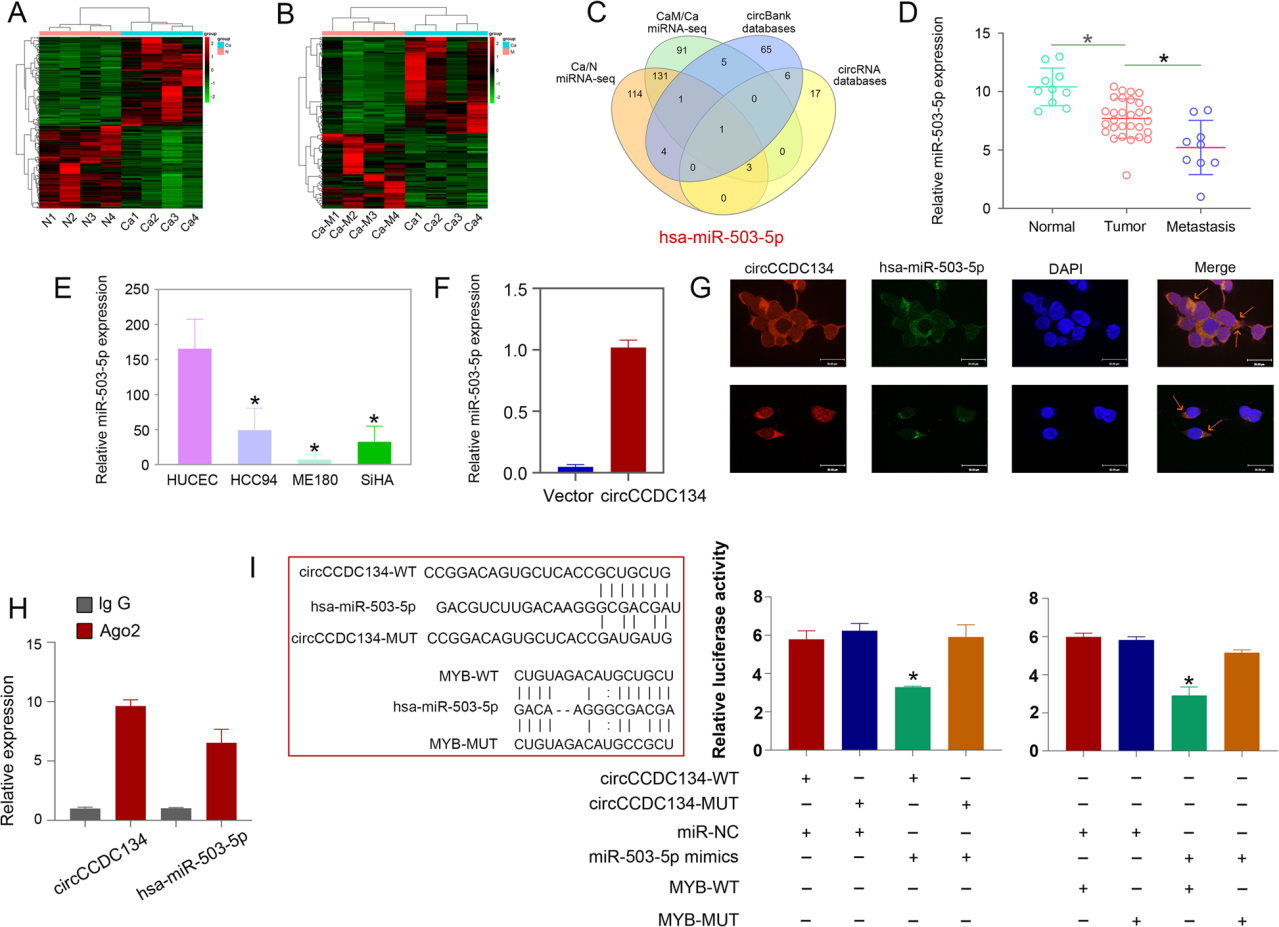
circCCDC134 acts as a miR-503-5p sponge to regulate the expression of MYB. **A** and **B** Heatmap of miRNA-seq data of CC tissues and metastatic tissues. **C**, **D** The expression of miR-503-5p was low in 29 tumour and 9 metastatic tissues and **E** CC cells. **F** circRNA pull-down assays showed that miR-503-5p could specifically bind circCCDC134, and **G**: RNA FISH assays revealed that circCCDC134 and miR-503-5p colocalized in the cytoplasm. **H** The RIP assay illustrated that circCCDC134 and miR-503-5p enrichment was increased in the Ago2 group. **I** The luciferase reporter gene assay showed that luciferase activity was significantly inhibited in cells cotransfected with the miR-503-5p mimic and wild-type luciferase reporter

Another key role of circRNAs is to sponge miRNAs and regulate downstream genes. To explore the miRNAs that could be sponged by circCCDC134, miRNA-seq data from CC tissues and metastatic tissues (Fig. 6A and B), the circBank database (http://www.circbank.cn/) and the circRNA database (https://circinteractome.nia.nih.gov/) were used. Among these data, only hsa-miR-503-5p was low in CC tissues and may be sponged by circCCDC134 (Fig. 6C). The qRT–PCR results revealed that the expression of miR-503-5p was low in the tumour and metastatic tissues (Fig. 6D) and CC cells (Fig. 6E). The circRNA pull-down assays showed that miR-503-5p could specifically bind circCCDC134 (Fig. 6F). The RNA FISH assays revealed that circCCDC134 and miR-503-5p colocalized in the cytoplasm (Fig. 6G). The RIP assay also illustrated that circCCDC134 and miR-503-5p enrichment was increased in the Ago2 group compared that in the IgG group (Fig. 6H). We investigated the role of miR-503-5p in the proliferation of CC cells by a colony formation assay. To assess the influence of miR-503-5p on cell migration and invasion, Transwell assays were used to detect the cell migration and invasion capacity. The results showed that the transfection of the miR-503-5p mimic resulted in a decrease in proliferation, migration and invasion ability in ME180 cells, and the function of miR-503-5p was rescued by the reintroduction of circCCDC134 (Supplementary Fig. 3A). Furthermore, we sought to identify the specific target gene of miR-503-5p via the miRDB, miRTarBase and TargetScan databases. According to the miRDB, miRTarBase and TargetScan database analysis, 90 target genes were found (Supplementary Fig. 3B). Combined with the si-circCCDC134 mRNA-seq data, 14 target genes were analysed (Supplementary Fig. 3C).

Next, we explored the OS (overall survival) and RFS (relapse-free survival) associated with these 14 genes in CC based on TCGA databases. The analysis revealed that MYB (Supplementary Fig. 3D), CD2AP, KPNA3, NUFIP2, and TLL1 (Supplementary Figs. 4 and 5) expression was closely related to CC OS and RFS. Since MYB is known to be associated with CC migration and invasion [26], we chose MYB for further study. The luciferase reporter gene assay consists of cloning both the wild-type and mutated forms of the 3’UTR of the miRNA predicted mRNA target downstream of a firefly luciferase reporter. The luciferase reporter gene assay showed that luciferase activity was significantly inhibited in HEK-293 T cells that were cotransfected with the miR-503-5p mimic and wild-type luciferase reporter (circCCDC134-WT and MYB-WT) compared with those in the control group (Fig. 6I). Moreover, the qPCR results showed that the expression of MYB was rescued by transfection with sh-circCCDC134 and anti-miR-503-5p (Supplementary Fig. 3E) or transfection with circCCDC134 and miR-503-5p mimics. These findings indicate that circCCDC134 can act as a sponge for miR-503-5p to upregulate MYB in CC cells.

### HIF1A as a downstream target of MYB

**Fig. 7:**
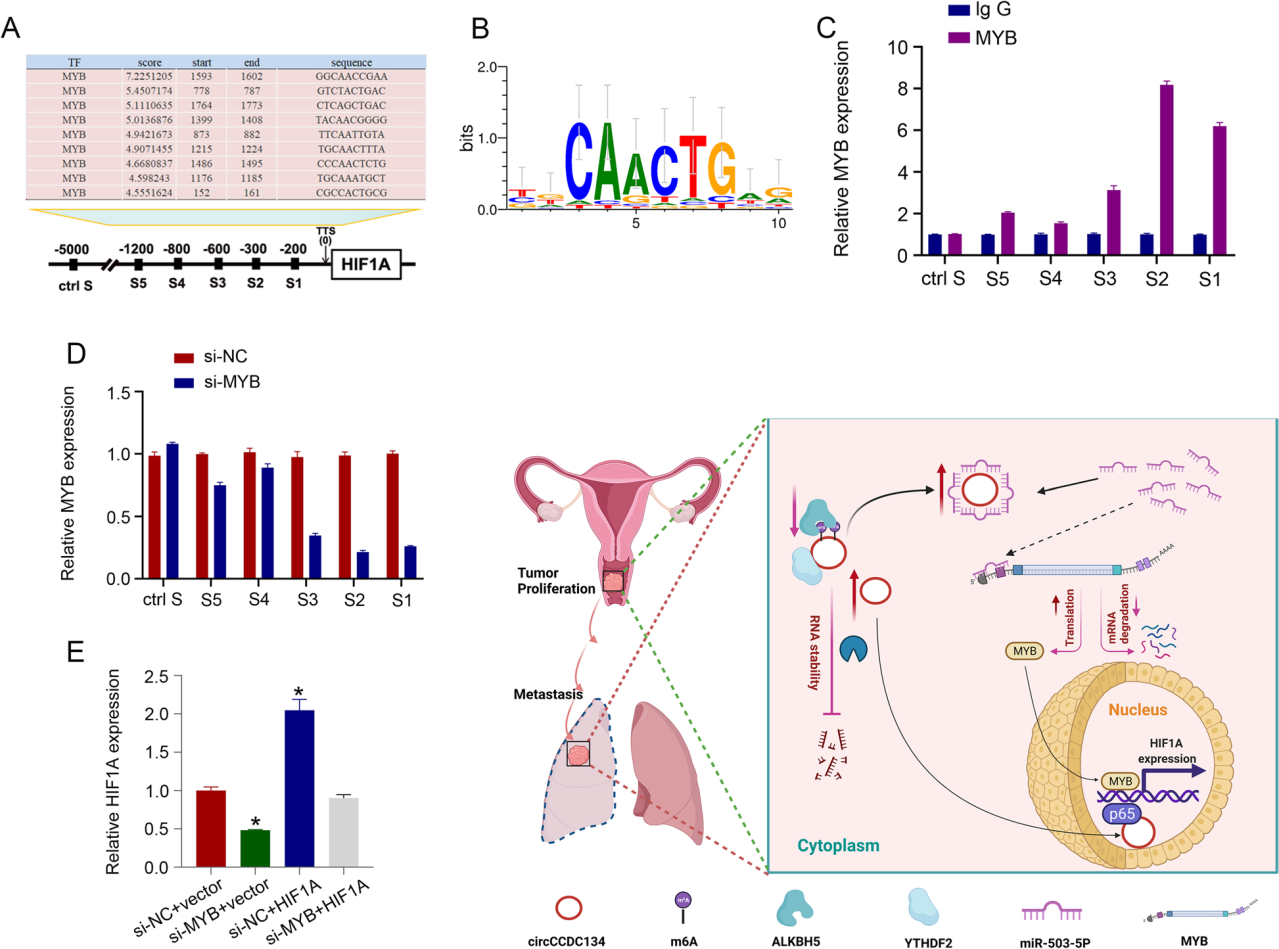
HIF1A is a downstream target of MYB. HIF1A is an important MYB target gene. **A** HIF1A is an important MYB target gene. **B** Motif analysis of the HIF1A transcription promoter site. **C** MYB was enriched in the region − 1200 to − 200 bp from the transcription start site of the HIF1A promoter. **D** MYB enrichment of the HIF1A promoter was reduced after MYB expression interference. **E** The expression of HIF1A was rescued by transfection with MYB. **F** Graphic Abstract

In previous studies, MYB was shown to be a very important transcription factor. To explore the downstream target of MYB, the JASPAR database (https://jaspar.genereg.net/) was used. Interestingly, HIF1A was an important MYB target gene (Fig. 7A). The motif analysis of the HIF1A transcription promoter site based on the high confidence of JASPAR is shown in Fig. 7B. The ChIP results showed that 1. MYB was enriched in the region − 1200 to − 200 bp from the transcription start site of the HIF1A promoter (Fig. 7C), and 2. MYB enrichment in − 1200 to − 200 bp from the transcription start site of the HIF1A promoter was reduced after MYB interference (Fig. 7D). Moreover, the qPCR results showed that the expression of HIF1A was rescued by transfection with MYB (Fig. 7E). These results demonstrate that MYB could stimulate HIF1A transcription.

## Discussion

Metastasis of cervical cancer is often the main cause of treatment failure, and prognosis is still unsatisfactory [28]. Therefore, the identification of new therapeutic targets to improve the prognosis of patients with this disease is needed. Mounting evidence indicates that circRNAs are stable, conserved and diverse and often exhibit tissue- or developmental stage-specific expression. Due to their abundance and high stability, circRNAs are suitable as novel clinical molecular markers, thereby providing new insights into the treatment of diseases.

In this study, circRNA-Seq was used to analyse dysregulated circRNA expression in CC, and one of the new molecules that attracted our attention was circCCDC134. To detect the features of circCCDC134, the following experiments were performed: 1. Sanger sequencing was used to validate the existence and structure of circCCDC134; 2. qRT–PCR with oligo (dT) revealed that circCCDC134 contained no poly-A tail; 3. RNase R and actinomycin D showed that circCCDC134 was more stable than linear CCDC134; and 4. according to the FISH and RNA fractionation assays, the fluorescence signal of circCCDC134 was found in both the nucleus and cytoplasm. Additionally, we revealed upregulated expression of circCCDC134 in CC metastasis tissues and cells using qRT–PCR. Our data indicate that the expression of circCCDC134 was significantly high in CC tissues, especially in metastatic tissues. The FISH and RNA fractionation assays suggested that circCCDC134 could play a regulatory role in both the nucleus and the cytoplasm. Then, we focused on the mechanism underlying the association between circCCDC134 and CC metastasis in terms of dysregulated expression and regulatory mechanisms.

N6-methyladenosine is the most abundant and reversible internal modification in mRNAs and circRNAs; recent studies have shown that m6A modification participates in circRNA expression. ALKHB5 is a member of the AlkB family and can remove m6A methylation from target RNAs and lead to lower m6A levels [16]. An increasing number of studies have found that ALKBH5 plays an important role in cancer progression. For example, ALKBH5 inhibits tumour growth and metastasis by abolishing the expression and activity of YAP in non-small cell lung cancer [29]. In the present study, the ChIRP-MS and RIP assays revealed that circCCDC134 cooperated with ALKBH5 and YTHDF2, and the qRT–PCR and actinomycin D assays showed that the expression and stability of circCCDC134 were decreased with ALKBH5 overexpression. These results show that circCCDC134 upregulation in CC was fine-tuned by ALKBH5-mediated m6A modification, which enhanced its stability in a YTHDF2-dependent manner. This type of m6A modification has been reported in previous studies investigating CC [30] and circular RNA expression in hepatocellular carcinoma [31], and our findings are consistent with these studies. Here, we first report that circCCDC134 is dominated by ALKBH5-mediated demethylation, followed by recognition and destabilization by YTHDF2, which is the mechanism by which circCCDC134 is dysregulated in CC metastasis. Our data provide novel evidence of circCCDC134 degradation in CC metastasis.

Recent studies have revealed that circRNAs can interact with RBPs and function as miRNA sponges to promote cancer metastasis. For instance, hsa_circ_0003258 promotes prostate cancer metastasis by complexing with IGF2BP3 and sponging miR-653-5p [32]. In our previous study, circACTN4 promoted intrahepatic cholangiocarcinoma proliferation and metastasis by acting as a molecular sponge of miR-424-5p and interacting with YBX1 to transcriptionally activate FZD7. In this study, we demonstrate that circCCDC134 exerts its function not only through a ceRNA mechanism but also by recruiting p65, ultimately stimulating HIF1A transcription and facilitating CC growth and metastasis. The functional analyses validated the role of circCCDC134 in promoting the proliferation and metastasis of CC cells both in vivo and in vitro. To explore the regulatory mechanism of circCCDC134, ChIRP-MS assays, RIP assays, miRNA-seq/mRNA-seq of CC tissues and metastatic tissues, mRNA-seq of ME180 cells with si-circCCDC134, ChIP-seq and ChIP–qPCR assays were applied. The results revealed that circCCDC134 recruited p65 to the nucleus and acted as a miR-503-5p sponge to regulate the expression of MYB in the cytoplasm, ultimately stimulating HIF1A transcription and facilitating CC growth and metastasis. Evidence indicates that a high expression of HIF1A is associated with a worse 5-year overall survival rate in cervical cancer [33], which is consistent with the findings of the current study. Our results confirmed that circCCDC134 plays a crucial role in CC metastasis and progression by interacting with p65 and regulating the expression of the miR-503-5p target gene MYB. Interestingly, we found that HIF1A was an important target gene for both p65 and MYB. These findings indicate that circCCDC134 plays a key role in enhancing HIF1A transcription.

## Conclusion

Our study was not free of limitations. First our results need to be validated in larger CC patient cohorts in terms of the overall survival prediction of circCCDC134. Second, we focused only on the roles of circCCDC134 in tumour proliferation and metastasis. More detailed studies are necessary to explore the impact of circCCDC134 on other malignant biological behaviours of CC cells, including angiogenesis and immune escape. Therefore, these unresolved limitations need to be addressed in future studies.

In summary, our findings highlight the attractive value of m6A demethylases in the expression of circCCDC134 and the key role of circCCDC134 in enhancing HIF1A transcription in the cytoplasm and nucleus. These findings shed light on novel molecular mechanisms and provide new insight into developing effective therapeutic strategies for CC metastasis.

## Supplementary Information

**Additional file 1: Supplementary Table 1.** Information concerning the clinical samples.

**Additional file 2: Supplementary Table 2.** Sequence information.

**Additional file 3: Supplementary Fig. 1.** (A and B): Melting curve and separation curve display the primers for circCCDC134 designed for the qRT–PCR experiments. (C): RNA fractionation assays showed that circCCDC134 was expressed in both the nucleus and cytoplasm of SiHA cells. (D and E): The mass spectrometry results of circCCDC134-binding proteins. (F): PPI analysis based on mass spectrometry data revealed that the ALKBH5 and YTHDF2 proteins could bind circCCDC134-MS2. Gene-specific m6A qPCR to detect the m6A methylation status of circCCDC134 was performed and the results indicated that an reduction of m6A methylation in the ALKBH5 overexpression group of (G): SiHA and (H): ME180 cell lines. (I): Patients with high ALKBH5 expression had a much longer overall survival in CC.

**Additional file 4: Supplementary Fig. 2.** (A): The expression of EFNA1 was lower in CC tissues. (B, C and D): The expression of KLF2, PER1 and COL5A3 was higher in CC tissues. The survival analysis based on these genes in CC based on TCGA databases revealed that EFNA1, KLF2, PER1 and COL5A3 expression was closely associated with CC overall survival.

**Additional file 5: Supplementary Fig. 3.** (A): Transfection of the miR-503-5p mimic resulted in a decrease in proliferation, migration and invasion ability in ME180 cells, and the function of miR-503-5p was rescued by the reintroduction of circCCDC134. (B): According to the miRDB, miRTarBase and TargetScan database analysis, 90 target genes were found. (C): Combined with the si-circCCDC134 mRNA-seq data, 14 target genes were analysed. (D): MYB was found to be the key target gene of miR-503-5p and was closely related to CC OS and RFS. (E): The qPCR results showed that the expression of MYB was rescued by transfection with sh-circCCDC134 and anti-miR-503-5p or transfection with circCCDC134 and miR-503-5p mimics.

**Additional file 6: Supplementary Fig. 4.** The survival analysis of 14 genes in CC based on TCGA databases. (A): Patients with high ANK3 expression had a much longer OS in CC. (B-G): Patients with low ANLN, CD2AP, KPNA3, NUFIP2, SEC24A or TLL1 expression had a better OS in CC. (H-M): OS analysis revealed that MYB CDK17, CCND2, ZBTB34, TSC22D2, TMEM245 and ZNF449 expression is not correlated with patient survival in CC.

**Additional file 7: Supplementary Fig. 5.** The RFS of 14 genes in CC based on TCGA databases. (A): Patients with high ANK3 expression had a much longer RFS in CC. (B-G): Patients with low ANLN, CD2AP, KPNA3, NUFIP2, SEC24A or TLL1 expression had a better RFS in CC. (H-M): The RFS analysis revealed that MYB CDK17, CCND2, ZBTB34, TSC22D2, TMEM245 and ZNF449 expression is not correlated with RFS in CC.

## Data Availability

The data used or analysed during the current study are available from the corresponding author upon reasonable request.

## Abbreviations

CC: Cervical cancer
circRNAs: Circular RNAs
m6A: N6-methyladenosine
ChIRP-MS: CircRNA pull-down and mass spectrometry
Chip-seq: Chromatin immunoprecipitation-seq
RIP: RNA binding protein immunoprecipitation
miRNA: MicroRNA
RBP: RNA-binding protein
TRAP: Tagged RNA affinity purification
ALKBH5: a-ketoglutarate-dependent dioxygenase AlkB homologue 5
YTHDF2: YTH N6-methyladenosine RNA binding protein 2
PPI: Protein-protein interaction networks
GO: Gene Ontology
KEGG: Kyoto Encyclopedia of Genes and Genomes
GSEA: Gene set enrichment analysis
EMT: Epithelial-mesenchymal transition
OS: Overall survival
RFS: Relapse free survival
