# Supplementary material for: ALKBH5-mediated m6A modification of circCCDC134 facilitates cervical cancer metastasis by enhancing HIF1A transcription
Source: J Exp Clin Cancer Res. 2022 Aug 26;41:261. doi: 10.1186/s13046-022-02462-7 (PMC9413927; doi:10.1186/s13046-022-02462-7)
Supplement: Supplementary file 1 — Additional file 1: Supplementary Table 1. Information concerning the clinical samples. [file 13046_2022_2462_MOESM1_ESM.docx]

| **Age(years)** |  |
| --- | --- |
| <45 | 20 |
| ≥45 | 26 |
| **Tumor size** |  |
| **<4 cm** | 26 |
| **≥4 cm** | 20 |
| **FIGO stages** |  |
| I–II | 29 |
| III–IV | 17 |

Information of the clinical samples
