# Supplementary material for: ALKBH5-mediated m6A modification of circCCDC134 facilitates cervical cancer metastasis by enhancing HIF1A transcription
Source: J Exp Clin Cancer Res. 2022 Aug 26;41:261. doi: 10.1186/s13046-022-02462-7 (PMC9413927; doi:10.1186/s13046-022-02462-7)
Supplement: Supplementary file 2 — Additional file 2: Supplementary Table 2. Sequence information. [file 13046_2022_2462_MOESM2_ESM.docx]

The information of sequence

| si-circCCDC134-1 | CGACCGCACAGAGCUCAAGTT  CUUGAGCUCUGUGCGGUCGTT |
| --- | --- |
| si-circCCDC134-2 | CACAGAGCUCAAGAGGUUUTT  AAACCUCUUGAGCUCUGUGTT |
| sh-circCCDC134 | CGACCGCACAGAGCTCAAG |
| si-P65 | siB09818131846-1-5, RiboBio,China |
| si-MYB | siG000004602A-1-5, RiboBio,China |
| anti-miR-503-5p | miR20002874-1-5, RiboBio,China |
| miR-503-5p mimic | miR10002874-1-5, RiboBio,China |

The sequence of primers

| circCCDC134-F | AATCGACCGCACAGAGCTCA |
| --- | --- |
| circCCDC134-R | GGTCCAGGGAGGTCCTCAAG |
| ALKBH5-F | CCAGCTATGCTTCAGATCGCCT |
| ALKBH5-R | GGTTCTCTTCCTTGTCCATCTCC |
| EFNA1-F | CTGTCTGAGAAGTTCCAGCGCT |
| EFNA1-R | CCACTGACAGTCACCTTCAACC |
| KLF2-F | CCAAGAGTTCGCATCTGAAGGC |
| KLF2-R | CCGTGTGCTTTCGGTAGTGGC |
| PER1-F | TCAACTGCCTGGACAGCATCCT |
| PER1-R | TCAGAGGCTGAGGAGGTGGTAT |
| COL5A3-F | GAGAGGAGAACTGGGCTTCCAA |
| COL5A3-R | TAGAGGTCCCACTTCTCCTGTC |
| HIF1A-F | TATGAGCCAGAAGAACTTTTAGGC |
| HIF1A-R | CACCTCTTTTGGCAAGCATCCTG |
| MYB-F | GGGAACAGATGGGCAGAAATCG |
| MYB-R | GCTGGCTTTTGAAGACTCCTGC |
| miR-503-5p-F | CCTATTTCCCATGATTCCTTCATA |
| miR-503-5p-R | GTAATACGGTTATCCACGCG |
| U6-F | ATTGGAACGATACAGAGAAGATT |
| U6-R | GGAACGCTTCACGAATTTG |
| GAPDH-F | GTCTCCTCTGACTTCAACAGCG |
| GAPDH-R | ACCACCCTGTTGCTGTAGCCAA |
