# Supplementary figures and images for: ALKBH5-mediated m6A modification of circCCDC134 facilitates cervical cancer metastasis by enhancing HIF1A transcription
Source: J Exp Clin Cancer Res. 2022 Aug 26;41:261. doi: 10.1186/s13046-022-02462-7 (PMC9413927; doi:10.1186/s13046-022-02462-7)

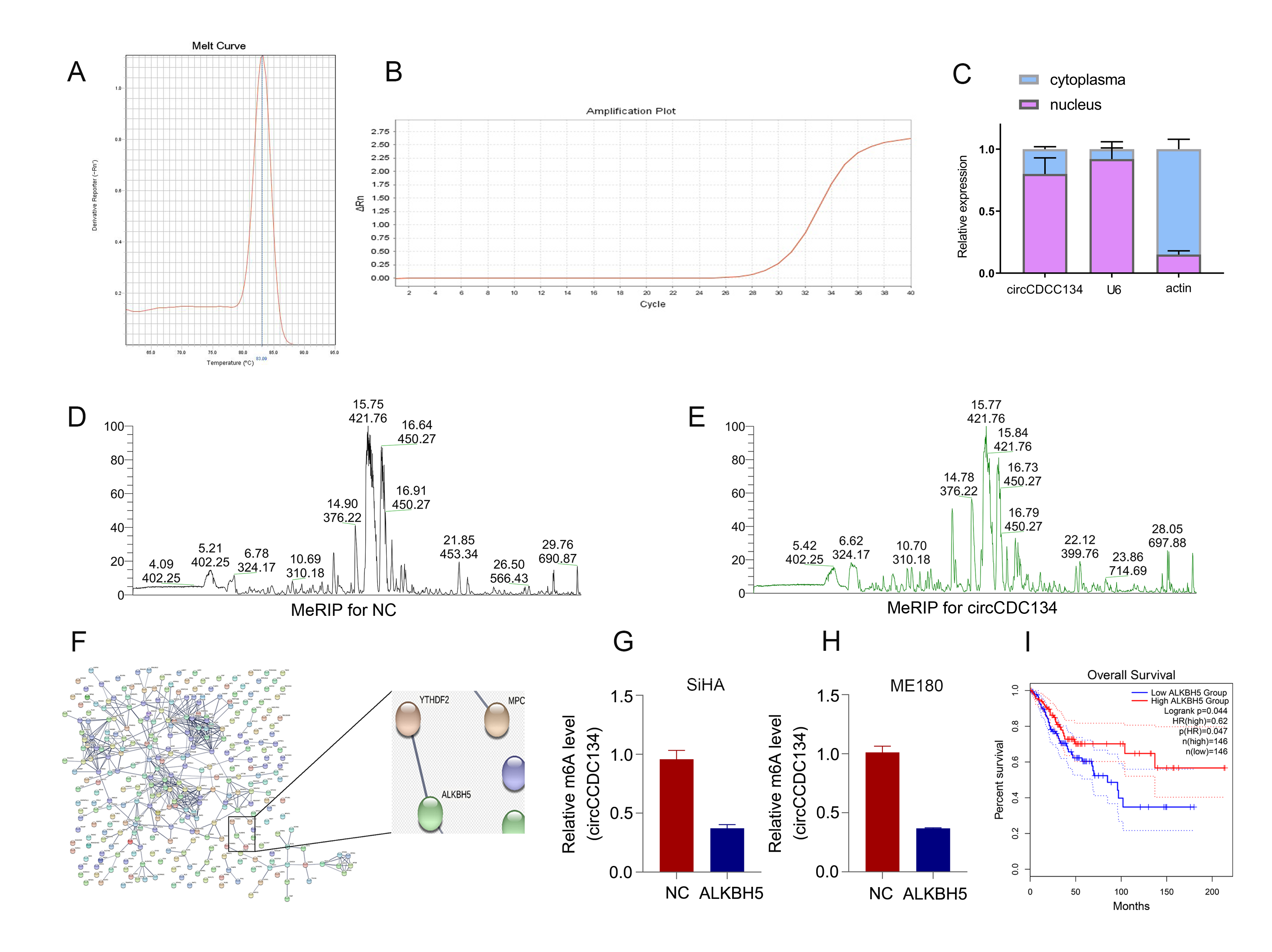

Supplement: Supplementary file 3 — Additional file 3: Supplementary Fig. 1. (A and B): Melting curve and separation curve display the primers for circCCDC134 designed for the qRT–PCR experiments. (C): RNA fractionation assays showed that circCCDC134 was expressed in both the nucleus and cytoplasm of SiHA cells. (D and E): The mass spectrometry results of circCCDC134-binding proteins. (F): PPI analysis based on mass spectrometry data revealed that the ALKBH5 and YTHDF2 proteins could bind circCCDC134-MS2. Gene-specific m6A qPCR to detect the m6A methylation status of circCCDC134 was performed and the results indicated that an reduction of m6A methylation in the ALKBH5 overexpression group of (G): SiHA and (H): ME180 cell lines. (I): Patients with high ALKBH5 expression had a much longer overall survival in CC. [file 13046_2022_2462_MOESM3_ESM.tif]

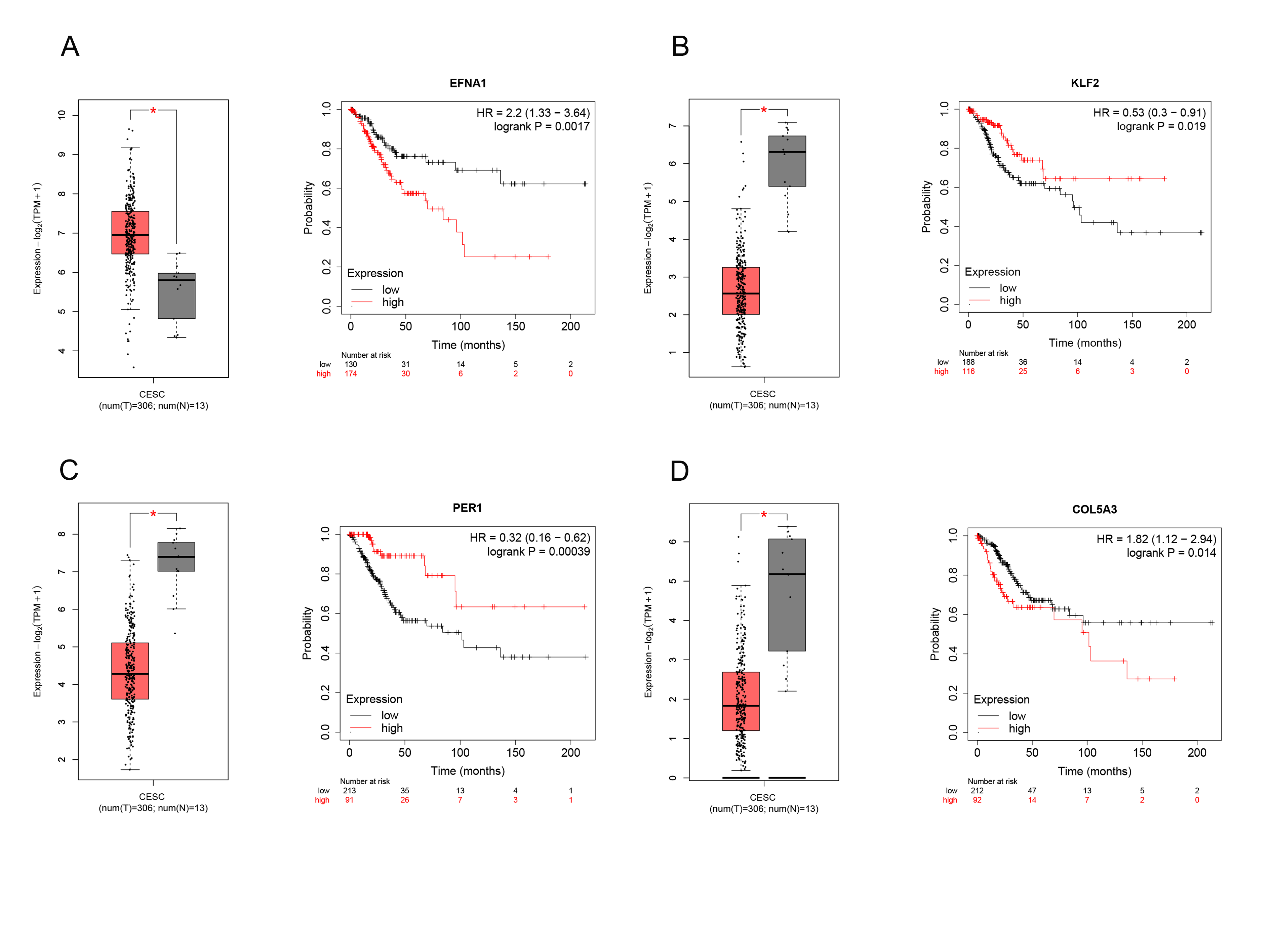

Supplement: Supplementary file 4 — Additional file 4: Supplementary Fig. 2. (A): The expression of EFNA1 was lower in CC tissues. (B, C and D): The expression of KLF2, PER1 and COL5A3 was higher in CC tissues. The survival analysis based on these genes in CC based on TCGA databases revealed that EFNA1, KLF2, PER1 and COL5A3 expression was closely associated with CC overall survival. [file 13046_2022_2462_MOESM4_ESM.tif]

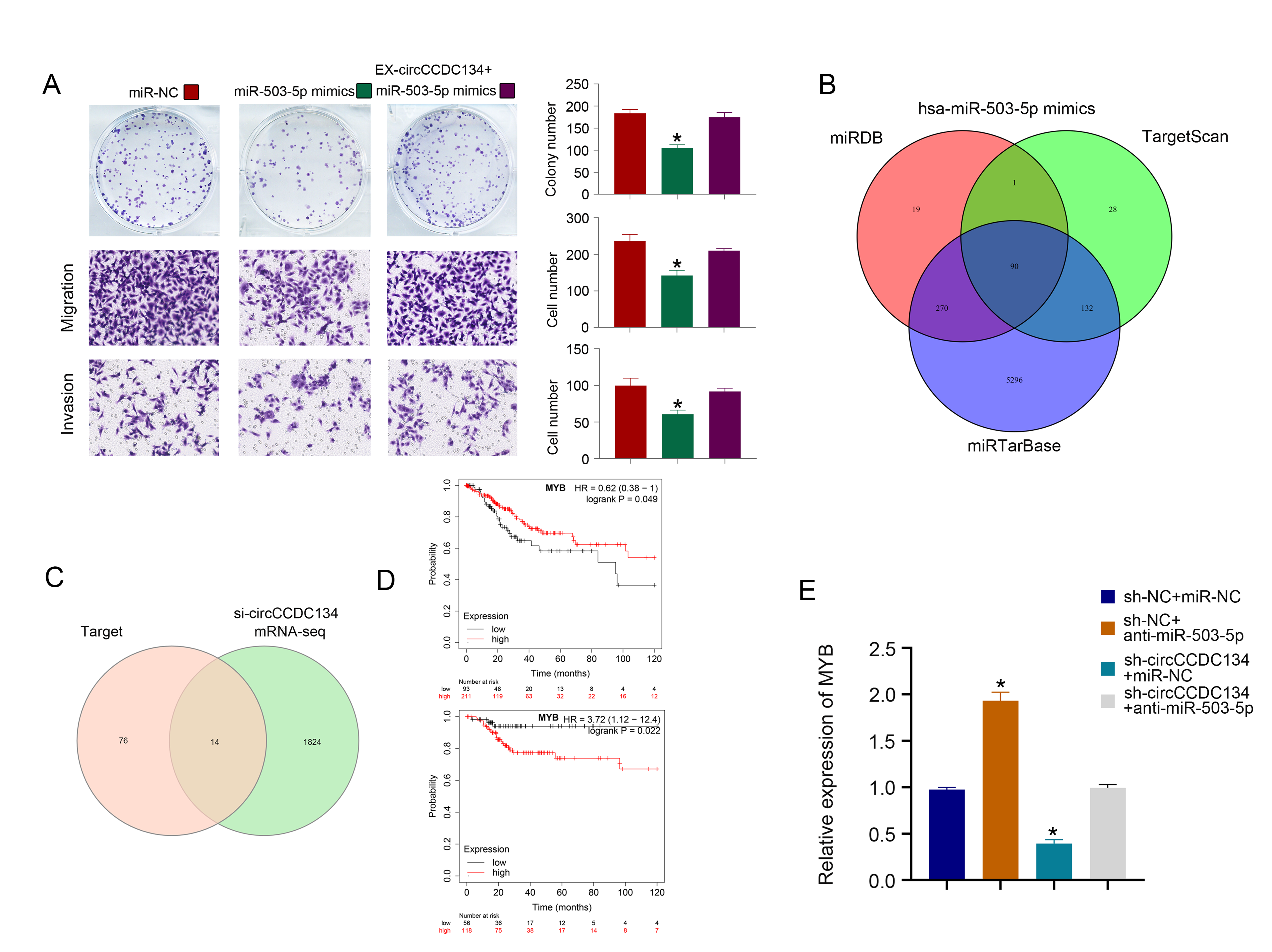

Supplement: Supplementary file 5 — Additional file 5: Supplementary Fig. 3. (A): Transfection of the miR-503-5p mimic resulted in a decrease in proliferation, migration and invasion ability in ME180 cells, and the function of miR-503-5p was rescued by the reintroduction of circCCDC134. (B): According to the miRDB, miRTarBase and TargetScan database analysis, 90 target genes were found. (C): Combined with the si-circCCDC134 mRNA-seq data, 14 target genes were analysed. (D): MYB was found to be the key target gene of miR-503-5p and was closely related to CC OS and RFS. (E): The qPCR results showed that the expression of MYB was rescued by transfection with sh-circCCDC134 and anti-miR-503-5p or transfection with circCCDC134 and miR-503-5p mimics. [file 13046_2022_2462_MOESM5_ESM.tif]

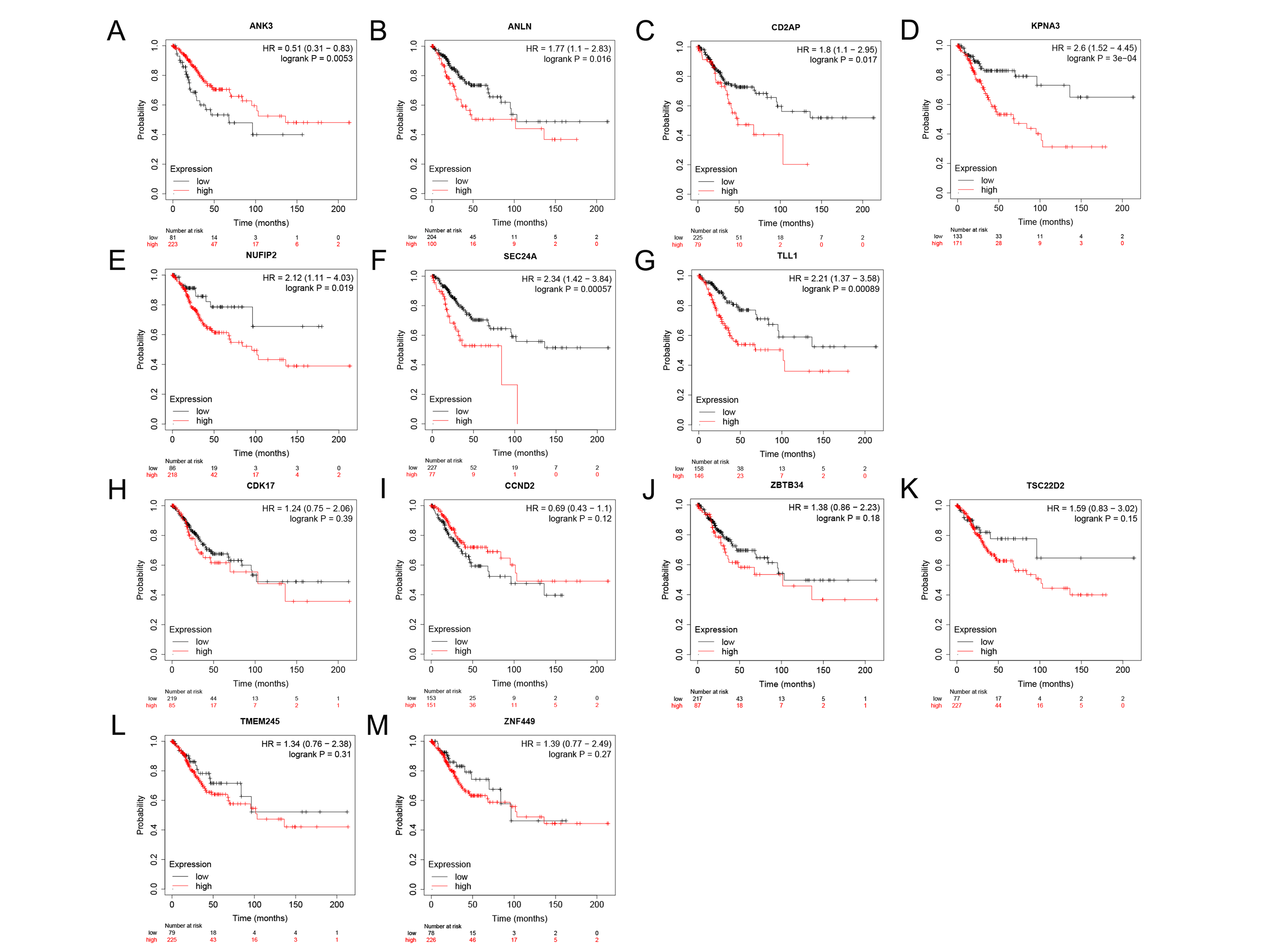

Supplement: Supplementary file 6 — Additional file 6: Supplementary Fig. 4. The survival analysis of 14 genes in CC based on TCGA databases. (A): Patients with high ANK3 expression had a much longer OS in CC. (B-G): Patients with low ANLN, CD2AP, KPNA3, NUFIP2, SEC24A or TLL1 expression had a better OS in CC. (H-M): OS analysis revealed that MYB CDK17, CCND2, ZBTB34, TSC22D2, TMEM245 and ZNF449 expression is not correlated with patient survival in CC. [file 13046_2022_2462_MOESM6_ESM.tif]

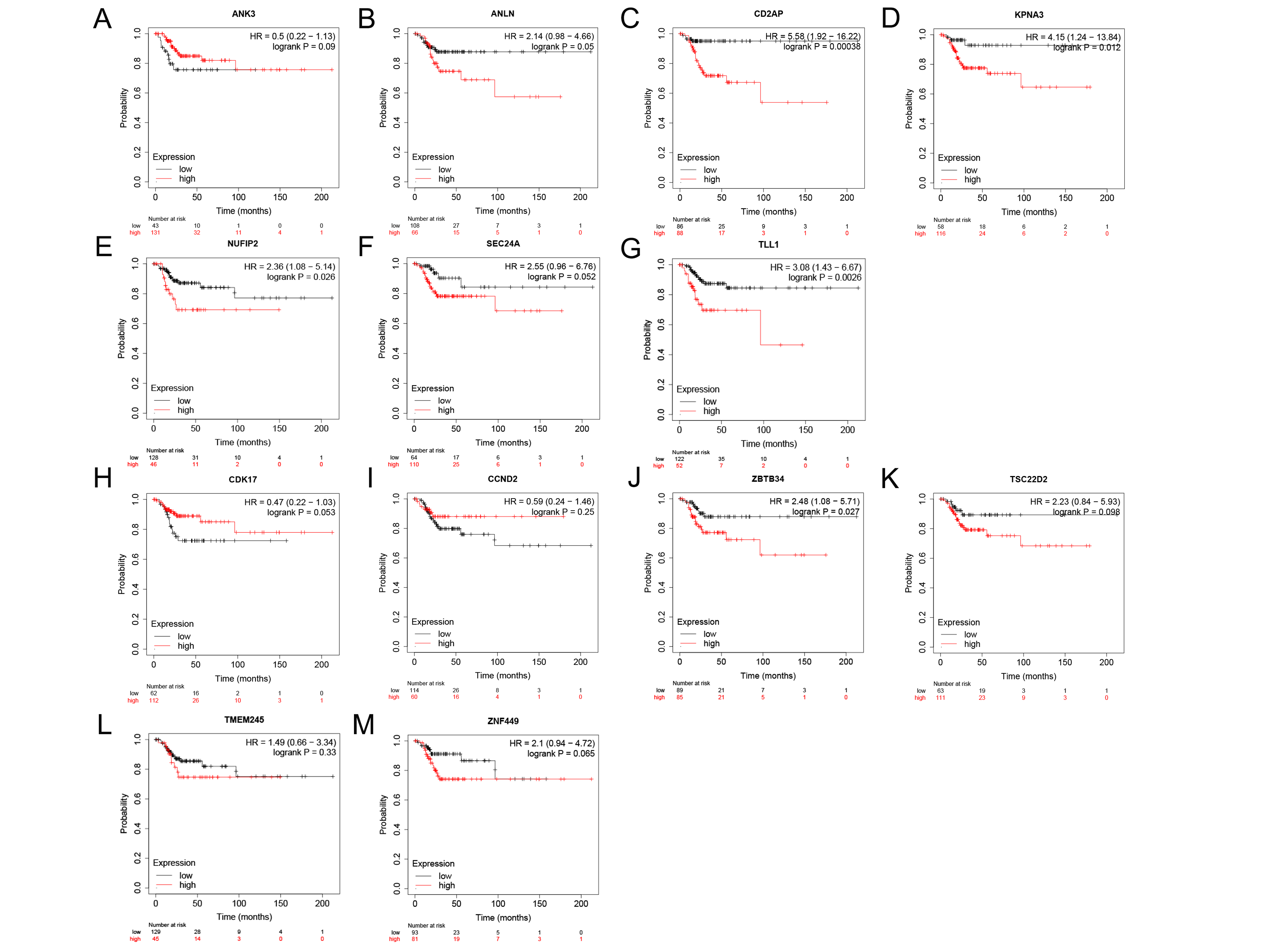

Supplement: Supplementary file 7 — Additional file 7: Supplementary Fig. 5. The RFS of 14 genes in CC based on TCGA databases. (A): Patients with high ANK3 expression had a much longer RFS in CC. (B-G): Patients with low ANLN, CD2AP, KPNA3, NUFIP2, SEC24A or TLL1 expression had a better RFS in CC. (H-M): The RFS analysis revealed that MYB CDK17, CCND2, ZBTB34, TSC22D2, TMEM245 and ZNF449 expression is not correlated with RFS in CC. [file 13046_2022_2462_MOESM7_ESM.tif]
